# The comparison of analgesic effects of various administration methods of diclofenac sodium, transdermal, oral and intramuscular, in early postoperative period in laparoscopic cholecystectomy operations

**DOI:** 10.12669/pjms.301.4140

**Published:** 2014

**Authors:** Sedef Gulcin Ural, Ozlem Yener, Hasan Sahin, Tuncer Simsek, Bahar Aydinli, Aysegul Ozgok

**Affiliations:** 1 Sedef Gulcin Ural, Department of Anesthesiology and Reanimation, Ankara Yuksek Ihtisas Education and Research Hospital, Ankara, Turkey.; 2 Ozlem Yener, Department of Radiology, Ankara Yuksek Ihtisas Education and Research Hospital, Ankara, Turkey.; 3 Hasan Sahin, Department of Anesthesiology and Reanimation, Medical Faculty, Canakkale Onsekiz Mart University, Canakkale, Turkey.; 4 Tuncer Simsek, Department of Anesthesiology and Reanimation, Medical Faculty, Canakkale Onsekiz Mart University, Canakkale, Turkey.; 5 Bahar Aydinli, Department of Anesthesiology and Reanimation, Ankara Yuksek Ihtisas Education and Research Hospital, Ankara, Turkey.; 6 Aysegul Ozgok, Department of Anesthesiology and Reanimationm Ankara Yuksek Ihtisas Education and Research Hospital, Ankara, Turkey.

**Keywords:** Diclofenac sodium, Laparoscopic cholecystectomy, Tramadol, Postoperative pain, Transdermal

## Abstract

***Objective: ***The aim of this study was to compare the efficacy of oral, intra muscular and transdermal diclofenac sodium for pain treatment in patients undergoing laparoscopic cholecystectomy, and their effect on postoperative opioid consumption.

***Methods: ***Following informed consent, 90 ASA I-II patients scheduled for laparoscopic cholecystectomy were randomized into three groups. Group PO got oral diclofenac sodium 1 hour before the operation, Group IM 75 mg diclofenac sodium intra muscular and Group TD diclofenac sodium patch 6 hours before the operation. Patients were not premedicated. Routine anaesthesia induction was used. After the operation in post anaesthesia care unit tramadol HCl infusion was delivered by intravenous patient controlled analgesia (iv PCA). Ramsey Sedation Score (RSS), Modified Aldrete’s Score System(MASS) and Visual Analog Scale Pain Score (VAS) was used for postoperative evaluation. Postoperative opioid consumption was recorded.

***Results: ***Demographic characteristics, intraoperative and postoperative hemodynamics of the patients were similar between groups. Postoperative VAS were lower at all time points in Group IM and Group TD than in Group PO. Lowest Postoperative RSS were in Group IM and the highest were in Group PO, and the difference between groups was significant. There was no significant difference in Postoperative MASS between groups. Postoperative tramadol consumption was statistically different between groups. Tramadol consumption in Group IM and Group TD was lower than Group PO. Postoperative nausea and vomiting was not observed. Local complications related to transdermal and intra muscular applications was not reported.

***Conclusion: ***In patients undergoing ambulatory laparoscopic cholecystectomy, a noninvasive application transdermal diclofenac sodium is as effective as intramuscular diclofenac sodium and can be preferred in postoperative pain treatment.

## INTRODUCTION

The first choice in the treatment of symptomatic gallbladder stone is laparoscopic cholecystectomy. Pain encountered following the laparoscopic cholecystectomy (LC) can be related to surgical manipulation, intraabdominal pressure applied during operation, irritation caused by CO_2_ administered intraperitoneally and irritation caused by bile leakage during the operation.^1^^,^^2^ Pain following LC is perceived in epigastrium, back and shoulders, and most frequently in upper abdomen.^3^ Pain following the operation is one of the most important factors causing morbidity and mortality, two causes prolonging the hospital stay.^4^

Pre-emptive analgesia can both decrease the severity and duration of pain and also can delay the pain.^5^ Opioids, non-steroidal anti-inflammatory drugs (NSAID) are frequently used for this purpose. The aim of this study was compare the analgesic efficacy of oral, intramuscular and transdermal forms of diclofenac sodium in postoperative pain treatment in patients that will undergo LC, and their effect on postoperative tramadol consumption. 

## METHODS

Following approval of local ethics committee and consent of the patients 90 ASA I-II patients scheduled for laparoscopic cholecystectomy were admitted to the study. The patients were double blindly randomized into three groups (Group PO n=30, Group IM n=30, Group TD n=30). Simple randomization was accomplished with a computer-generated sequence of numbers and sealed envelopes were used to allocate patients into 3 groups. Patients with peptic ulcer and gastritis diagnosis, who have allergic reactions to study drugs, with serious liver and/or renal dysfunctions, pregnant women and ASA III and above patients were excluded from the study.

Routine monitorization (electrocardiography, non-invasive blood pressure and peripheral oxygen saturation) was performed to patients taken to the operation room without premedication. Anesthesia induction was performed with 6 mg^-1^kg^-1^ thiopental sodium, 1,5 mcg^-1^kg^-1^ fentanyl and 0,6 mg^-1^kg^-1^ rocuronium bromide and %50/%50 O2/air mixture containing sevoflurane (1-1,2 MAC) was used for maintenance. Diclofenac sodium retard tablet 75 mg oral was administered to Group PO one hour before the surgery; post-incisional 75 mg diclofenac sodium ampoule IM was administered to Group IM and diclofenac sodium patch was applied 6 hours before the surgery to Group TD. Tramadol was started with iv patient controlled analgesia method to patients before they were taken to the recovery room (bolus dose 10 mg, lock-out time 10 minute, basal infusion rate 10 mg^-1^hour^-1^). Ramsey Sedation Scale (RSS), Modified Aldrete Score (MASS) and Visual Analogue Scale Pain Score (VAS) of 0-10 (0= no pain and 10= worst imaginable pain) of the patients were evaluated in post operative 0, 15, 30 and 60^th^ minutes. Side effects such as nausea and vomiting were recorded with the opioid doses administered additionally.

***Statistical Analysis: ***Analysis of the data was performed in SPSS (Statistical Package for Social Science) for Windows 16.0.0 software package. Chi-square test was used in cross tables; average of ages of 3 groups, systolic arterial pressure (SAP), diastolic arterial pressure (DAP), heart rate (HR), peripheral O_2_ saturation, Aldrete scores, mean push count were evaluated with One Way Variance Analysis (One-Way ANOVA); Kruskal-Wallis tests were used for VAS and Ramsey scores; SAP, DAP within the same group, whether HR and peripheral oxygen saturation differed between 7 different periods were evaluated in parametric data with Variance Analysis with Repeated Measurements (ANOVA with repeated measurements) and in non-parametric data with Friedman test. The pair that caused the difference was determined with Post hoc multi comparison test following ANOVA, Variance Analysis with Repeated Measurements, Kruskal-Wallis and Friedman tests.

## RESULTS

**Fig.1:**
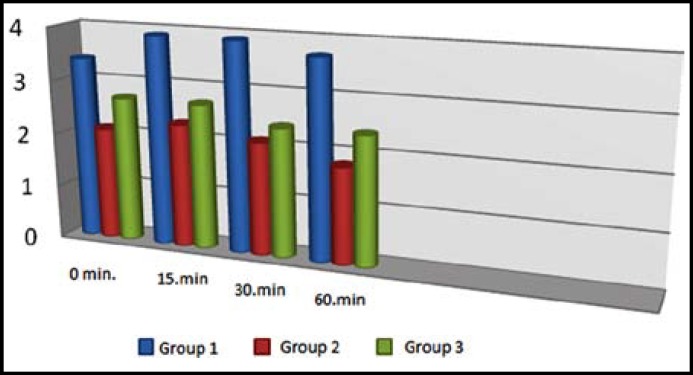
VAS scores according to study groups in follow-up periods

Groups were considered to be similar with regards to demographic data and intra-operative follow-up (Table-I). Post operative VAS pain scores were significantly lower in the 30th and 60th minutes in Group TD and in 60^th^ minute in Group IM when compared with the 15th minute scores (p<0.05). When VPS pain scores of all groups were evaluated VPS pain scores at 0 minute in Group IM and Group TD was found to be lower than Group PO. When VPS pain scores of all the follow-up periods were compared Group IM and Group TD had lower VPS values than Group PO (Table-II) (Fig.1). Group PO was the group that had the highest VPS values in all of the follow-up periods.

When intra-group bolus counts were considered with regards to additional medicine requirement, Group PO was statistically higher with 6 or more push counts (p<0.001) (Table-III). When inter-group additional medicine requirements were compared, the requirement was the lowest in Group IM and highest in group PO (P=0.038<0.05). Therefore opioid dose decrease is best provided in Group IM (Table-IV).

Post operative RSS were the lowest in Group IM and the highest in Group PO; inter-group difference was significant (p<0.05). Inter-group statistical differences were not detected in terms of nausea and vomiting.

## DISCUSSION

In this study, analgesic efficacies of transdermal, oral and intramuscular forms of Diclofenac sodium following LC were compared. A significant decrease in post operative VAS values was discovered in Group IM and Group TD when compared with the Group PO. Although post operative tramadol consumption was lower in Group IM no significant difference was observed from Group TD. The best opioid consumption decrease was observed in Group IM.

**Table-I:** Demographic characteristics of the groups

*Variables*	*Group PO (n=30)*	*Group IM (n=30)*	*Group TD (n=30)*	*P*
Age	44.9+12.3	46.8±14.0	46.9±10.9	0.781
Sex (M/F)	11/19	11/19	11/19	1.000
Comorbid Disease	6	7	10	0.468
Hypertension	6	6	6	1.000
Diabetes Mellitus	1	0	3	0.160
Cronic obstructive pulmonary disease	2	1	3	0.585

**Table-II:** VAS scores according to study groups during follow-up periods (Inter-groups).

*Follow-up periods*	*Group PO(n=30)*	*Group IM(n=30)*	*Group TD(n=30)*	*p*
0^th^ minute	3.4+1.0	2.1+0.6^a^	2.7+1.3^a,b^	0.000
15^th^ minute	3.9+1.2	2.3+1.0^c^	2.7+1.2^c^	0.000
30^th^ minute	3.9+1.2	2.1+0.8^d^	2.4+1.3^d^	0.000
60^th^ minute	3.7+1.1	1.8+0.6^e^	2.4+1.5^e^	0.000

a: Significant difference with Group PO in post operative 0th min. (p<0.001)

b: Significant difference with Group IM in post operative 0th min (p<0.001)

c: Significant difference with Group PO in post operative 15th min (p<0.001)

d: Significant difference with Group PO in post operative 30th min (p<0.001)

e: Significant difference with Group PO in post operative 60th min (p<0.001)

**Table-III:** Inter-group case distribution according to bolus tramadol requirement

*Push Count*	*Group PO(n=30) *	*Group IM (n=30) *	*Group TD(n=30) *
0 bolus	2	10	7
1 bolus	2	8	8
2 boluses	0	7	3
3 boluses	2	3	7
4 boluses	3	2	4
5 boluses	5	0	1
6 boluses	16 *	0	0
Total patient number	30	30	30

P=0.000<0.001, *: Patients in need of 6 or above pushes of additional medicine

**Table-IV:** Intra-group distribution according to bolus tramadol requirement

*Patient Number *	*Group PO(n=30)*	*Group IM(n=30)*	*Group TD(n=30)*	*Total*
Not bolused	2	10	7	19
Bolused	28	20	23	71

Post operative pain management is extremely important in ambulatory surgeries such as LC. Approximately 80% of the patients feel post operative pain following a surgery.^6^ Pain after the surgery peaks in the first hours and is controlled in the following hours and gradually decreases in the 2nd-3rd post operative days.^7^^,^^8^ According to the result of our study transdermal diclofenac sodium, a noninvasive practice in early period pain management in ambulatory surgeries such as LC can provide efficient analgesia as much as IM diclofenac sodium.

In the studies performed by Predel et al. and Galer et al. they showed that diclofenac sodium transdermal provided sufficient pain management without any side effects in pains developed related to acute injuries.^9^^,^^10^ Different forms of diclofenac sodium (oral, rectal, intravenous and intra muscular) were used in pain management following post operative laparoscopy in the studies found in literature.^11^^-^^13^ In the study Karabayirli et al. detected that transdermal form in pain management following the laparoscopic surgery is as much efficient method as im form.^14^ Rahimi et al. used transdermal diclofenac sodium in myalgia treatment related to succinylcholine use in the caesarean section and found it efficient.^15^

Alessandri F et al. administered transdermal diclofenac sodium into the incision area in postoperative period to a study group which is conducted on 120 patients undergoing gynecological surgery, they also gave placebo treatment to a second group in the same study. They have observed in the study group who had trandermal diclofenac sodium in postoperative period that there was significantly less analgesic consumption and a shorter duration of hospital discharge ^16^. In our study we observed that transdermal and intramuscular diclofenac sodium administered in preoperative period has similar analgesic effects. However we think that transdermal administration is much more comfortable and safe method for patients. Because intramuscular injection could make a disturbing pain. Bhaskar et al. compared analgesic effects of 100 mg oral diclofenac sodium with 100 mg transdermal diclofenac in 20 patients receiving orthodontic treatment and they found similar analgesic effects for both application but they have advised transdermal application for it’s comfort and much more less complications ^17^. In our study we observed oral diclofnac sodium provides less analgesic effect and causes increase in postoperative tramadol consumption when compared with IM. and transdermal application. Furthermore, we believe that the use of oral diclofenac sodium may increase gastrointestinal complaints in patients already stressful in preoperative period. Predel et al. compared analgesic effectiveness of placebo with transdermal application in a study of 120 patients admitted to the emergency department for blunt soft tissue injury and they have stated that transdermal application is an effective and reliable method ^18^. In a further study, Brühlmann et al. evaluated effectiveness of transdermal diclofenac sodium on 103 patients with knee osteoarthritis and reported that transdermal diclofenac sodium application to the placebo group is an effective and reliable method ^19^.

 In the study performed by Yanchick et al. 274 patients with acute knee strain were divided into two groups as placebo and diclofenac sodium patch groups and analgesia levels were followed up for 7 days. The earliest pain decrease in the first 3 post operative hours was reported as 1.27 hours when compared with the placebo group.^20^ Time needed to reach maximum efficiency for diclofenac sodium patch was 5.4+3.7 hours. We also think that application of diclofenac sodium patch 6 hours before surgery is effective in early post operative pain control. Since we restricted the pain follow-up with one hour due the short recovery room stay of patients in post operative period we can't comment on the long period effectiveness of transdermal diclofenac sodium use.

## CONCLUSION

In the study where analgesic effectiveness of 3 different forms of diclofenac sodium were compared in early period pain management we determined that transdermal form provided as efficient analgesia as IM form and decreased opioid consumption. We consider that the preference of diclofenac sodium in and patch forms in early period pain management in ambulatory cases such as LC will increase patient's comfort and will reduce side effect profile by decreasing additional opioid requirement.
